# Prevalence of Thyroid Disorder in Gallstone Disease Patients: A Cross-Sectional Study

**DOI:** 10.7759/cureus.52422

**Published:** 2024-01-17

**Authors:** Seema R Sinha, Prem Prakash

**Affiliations:** 1 Biochemistry, Indira Gandhi Institute of Medical Sciences, Patna, IND; 2 General Surgery, Indira Gandhi Institute of Medical Sciences, Patna, IND

**Keywords:** gallstone disease (gsd), subclinical hypothyriodism, dyslipidemia, hyperthyroidism, clinical hypothyroidism

## Abstract

Background

Gallstone disease (GSD) is one of the most common disorders involving the biliary system. The three types of gallstones include pigment, cholesterol, and mixed stones. Studies have suggested a potential association between thyroid dysfunction and lipid pathogenesis, which influences bile composition. A higher prevalence of thyroid disorders may have an impact on the management of GSD patients. This study aimed to assess the prevalence of thyroid disorders and associated dyslipidemias among individuals diagnosed with GSD.

Methodology

This cross-sectional, observational study was conducted among 180 eligible patients with a mean age of 47.72 ± 15.29 years. This study included 56 (31%) male and 124 (69%) female patients admitted to the Department of General Surgery, Indira Gandhi Institute of Medical Sciences, in eastern India. A diagnosis of GSD was established based on a radiological investigation (ultrasonography) and was included in the study. A thyroid profile test, a liver function test, and a lipid profile test were done for all patients. Patients with a previous surgical history of thyroid disease and known cases of hypothyroidism taking thyroxin supplements for treatment, as well as uncontrolled diabetes mellitus and hypertension, were excluded from the study. Relevant data were collected and statistically analyzed.

Results

Among the 180 patients, 122 (67.77%) were euthyroid, 35 (19.44%) had subclinical hypothyroidism, 20 (11.11%) had clinical hypothyroidism, and three (1.66%) had hyperthyroidism. Out of a total of 55 hypothyroidism patients, 37 (67.27%) had dyslipidemias.

Conclusions

The prevalence of hypothyroidism in GSD was 30%, with a female predominance. Hypothyroidism is a specific risk factor for cholelithiasis, and all patients with GSD who have dyslipidemia should be evaluated for thyroid dysfunction.

## Introduction

Gallstone disease (GSD) is one of the most common pathology of the biliary system. There are three types of gallstones, namely, pigment (brown/black), cholesterol, and mixed stones. In Asia, 85% of gallstones are pigment stones; however, in the United States, 80% are cholesterol or mixed stones [1]. Moreover, there is a high prevalence of cholesterol gallstones in the north Indian population, which are found in both the gallbladder and common bile duct (CBD). On the other hand, individuals from south India exhibit a higher prevalence of pigment gallstones, which are seen in both the gallbladder and the CBD [2].

Cholesterol is not a water-soluble substance. The solubility of cholesterol in bile depends upon the type and concentration of phospholipid and bile acid present in bile. Phospholipid-formed micelles maintain cholesterol in a stable thermodynamic state. When bile acid concentrations are low or supersaturated with cholesterol, it results in the formation of unstable unilamellar phospholipid vesicles, which may serve as nuclei for cholesterol crystals and lead to stone formation. Other causes of stone formation include bile stasis caused by dysfunction of the sphincter of Oddi, biliary sludge, bile saturation in the gallbladder, and a change in the chemical disproportion of bile [3].

Hypothyroidism is one of the important medical conditions that cause the development of GSD in adult patients [4]. Over the past decade, there has been considerable debate regarding the potential association between thyroid conditions and the development of GSD. According to a study by Rizos et al., there may be an association between thyroid gland dysfunction and the pathogenesis of lipids, which influences the composition of bile [5].

Studies by Tombisana et al., Raghuvanshi et al., and Laukkarinen et al. have demonstrated that patients with hypothyroidism have decreased biliary flow due to delayed gallbladder emptying, which plays an important role in the development of GSD [1,6,7]. Few studies have examined patients with GSD to determine the occurrence of previously undiagnosed thyroid disorder, with the results suggesting a potential association between hypothyroidism and GSD. The diagnosis and management of patients with GSD may be influenced by the increased frequency of thyroid dysfunction [8-11].

The objective of this study was to assess the prevalence of thyroid disorders and dyslipidemias among individuals diagnosed with GSD.

## Materials and methods

This cross-sectional, observational study was conducted at the Department of Biochemistry and Department of General Surgery, Indira Gandhi Institute of Medical Sciences, Patna, India, among 180 patients from January 2023 to November 2023, after obtaining approval from the Institutional Ethics Committee (approval number: 6233/IEC/IGIMS/2023). Informed consent from all participants was obtained once the study’s procedures were explained in their local language. Patients belonging to the age group of 18-80 years with GSD diagnosed by radiological investigation (ultrasonography) were selected for inclusion in the study.

The exclusion criteria included patients with a previous history of surgery for thyroid disorder and taking thyroxin, as well as patients with uncontrolled diabetes mellitus and hypertension, main pancreatic duct stones, cholangitis, and pregnancy. Patients taking drugs that could cause hypothyroidism (such as amiodarone, lithium, antidepressants, phenytoin, interferon, and imatinib) or the development of gallstones (such as estrogen/oral contraceptive pills, fenofibrate, and gemfibrozil) were not included in the study.

Study procedure

All consecutive patients diagnosed with GSD were enrolled in the study as per the inclusion and exclusion criteria during the visit to the outpatient department. A thorough clinical evaluation was performed. The demographic profiles of all patients were recorded. Routine baseline blood investigations were conducted, including a complete blood count, lipid profiles, liver function tests, and kidney function tests. A venous blood sample was collected after overnight fasting and sent to the Biochemistry Laboratory for investigation of the patient’s thyroid profile using the chemiluminescence technique on the Architech Abbott i2000SR. The thyroid status of the patients, whether euthyroid, hypothyroid, or hyperthyroid, was documented.

Patients with serum thyroid-stimulating hormone (TSH) levels between 0.5 and 4.9 mIU/L with normal T3 and T4 levels were considered euthyroid. Subclinical hypothyroidism was defined as serum TSH levels between 5 and 10 mIU/L with normal T3 and T4 levels. TSH concentrations greater than 10 mIU/L were considered clinical hypothyroidism [12].

Hypothyroidism was considered the dependent variable. Independent variables included age (ranging from 18 to 80 years), sex (male or female), comorbidities (hypertension, diabetes), sonographic findings, and thyroid profile (T3, T4, TSH).

Statistical analysis

All the data were collected and entered in an MS Excel spreadsheet (Microsoft Corp., Redmond, WA, USA), analyzed, and expressed as percentages. Data on quantitative characteristics are expressed as mean ± SD. Data on qualitative characteristics are expressed as percentages or absolute numbers as indicated. The chi-square test was used to compare groups with nominal data, whereas analysis of variance was used for continuous data. The statistical analysis was conducted using STATA version 17 for Windows (StataCorp., College Station, TX, USA). A p-value <0.05 was considered statistically significant.

## Results

**Figure 1:**
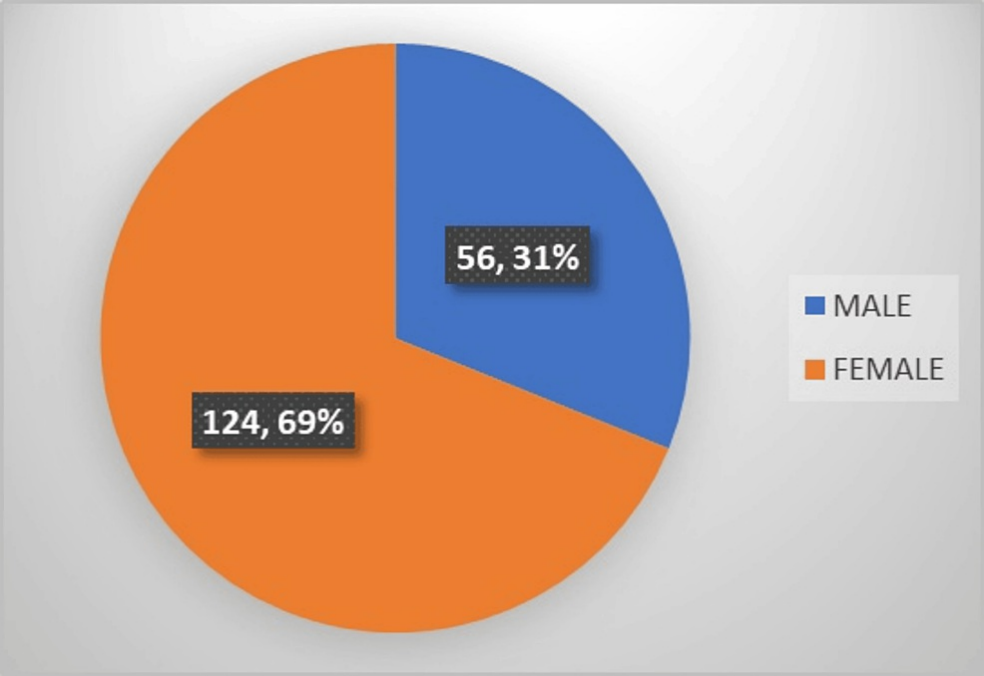
Pie chart showing sex distribution among participants with gallstone disease.

A total of 180 patients were included in this study. The participants’ ages ranged from 18 to 80 years, with a mean age of 47.72 ± 15.29 years. There were 56 (31%) male and 124 (69%) female patients in this study (Figure 1).

**Figure 2:**
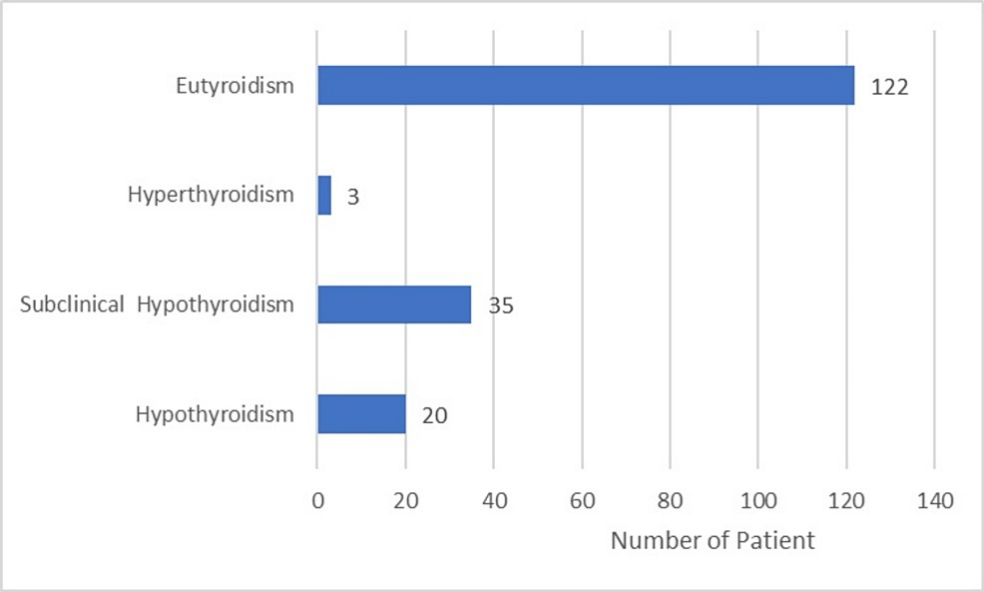
Bar chart showing the distribution of thyroid disorder.

**Figure 3:**
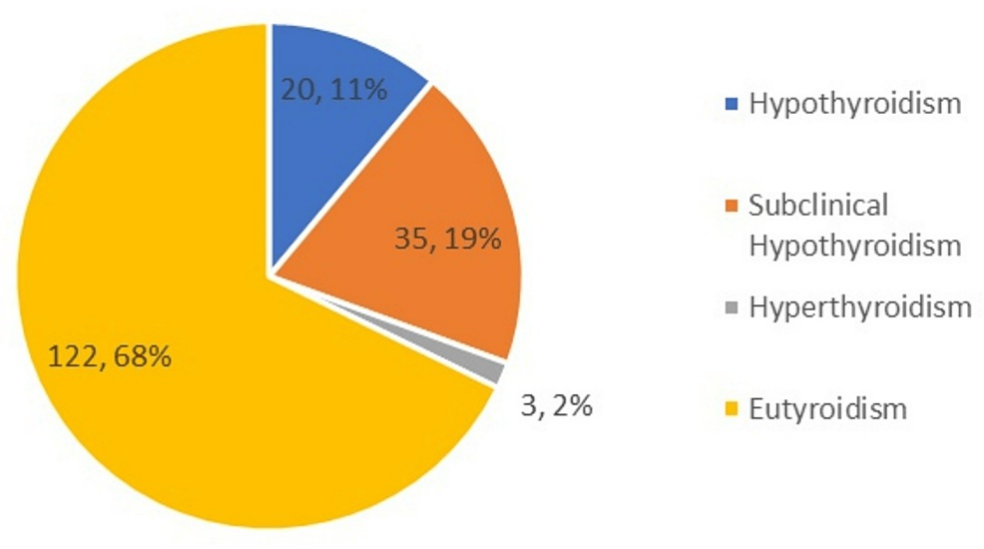
Pie chart showing the number and percentage of thyroid status among patients with gallstone disease.

The most common comorbidity was hypertension, seen in 15 (8.33%) patients, followed by diabetes mellitus in 10 (5.55%) patients. Twenty (11.11%) patients were found to have clinical hypothyroidism, 35 (19.44%) patients had subclinical hypothyroidism, three (1.66%) patients had hyperthyroidism, and the remaining 122 (67.77%) patients were found to be euthyroid (Figures 2, 3).

Overall, 14 of the 20 patients with overt hypothyroidism were female, while only six were male. Out of a total of 35 patients diagnosed with subclinical hypothyroidism, 25 were female and 10 were male. The predominant age group was 40-49 years (Table 1).

**Table 1:** Age and gender distribution of patients with gallstone disease. The data are presented as n (%).

Age (years)	Male	Female	Total
18–29	6 (3.33%)	12 (6.66%)	18
30–39	14 (7.77%)	24 (13.33%)	38
40–49	15 (8.33%)	35 (19.44%)	50
50–59	12 (6.66%)	35 (19.44%)	47
60–69	6 (3.33%)	15 (8.33%)	21
70–80	3 (1.66%)	3 (1.66%)	6
Total	56 (31.11%)	124 (68.88%)	180

Lipid profile and liver function tests were done for all patients. It was observed that 16 (8.88%) patients had low albumin levels, while seven (3.88%) had high direct bilirubin levels. Of the 20 clinical hypothyroidism patients, 12 (29.26%) had a higher level of serum cholesterol and 13 (28.88%) had high levels of triglyceride (TG). Of the 35 patients with subclinical hypothyroidism, 25 (60.97%) had raised cholesterol levels and 26 (57.77%) had high TG. Of the 122 patients with euthyroid status, four (9.75%) had high cholesterol and six (13.33%) had high TG (Table 2).

**Table 2:** Demographic and laboratory parameters. The data are presented as n (%). P-values <0.05 are considered statistically significant.

Variables	Clinical hypothyroidism	Subclinical hypothyroidism	Hyperthyroidism	Euthyroid	P-value
N (%)	20 (11.11%)	35 (19.44%)	3 (1.66%)	122 (67.77%)	NA
Sex					
Male	6	10	3	40	NA
Female	14	25	0	82	
Albumin					
Low	5 (31.25%)	3 (18.7%)	0	8 (50.0%)	0.023
Normal	15 (9.14%)	32 (19.5%)	3 (18.2%)	114 (69.5%)	
Direct bilirubin					
Normal	19 (10.98%)	31 (17.9%)	3 (1.73%)	120 (69.3%)	0.0023
high	1 (14.2%)	4 (57.14%)	0	2 (28.5%)	
Alkaline phosphatase					
Normal	19 (11.17%)	29 (17.05%)	3 (1.76%)	119 (70%)	0.79
High	1 (10%)	6 (60%)	0	3 (30%)	
Cholesterol					
Normal	8 (5.75%)	10 (7.19%)	3 (2.15%)	118 (84.89%)	0.012
High	12 (29.26%)	25 (60.97%)	0	4 (9.75%)	
Triglycerides					
Normal	7 (5.18%)	9 (6.66%)	3 (2.22%)	116 (85.92%)	0.032
High	13 (28.88%)	26 (57.77%)	0	6 (13.33%)	
Low-density lipoprotein					
Normal	8 (5.67%)	13 (9.21%)	3 (2.12%)	117 (82.97%)	0.80
High	12 (30.76%)	22 (56.41%)	0	5 (12.82%)	

## Discussion

This study aimed to evaluate the prevalence of thyroid disorders and associated dyslipidemias among individuals diagnosed with GSD to determine the significance of thyroid profiles as a contributory component and whether they should be included in regular examinations for these individuals. GSDs are one of the most common conditions encountered by surgeons, with an incidence ranging from 5% to 30%. The predisposition to gallstone formation increases with increasing age, parity, dyslipidemia, and hypothyroidism. The possible link between GSD and hypothyroidism disorder has been an area of interest for researchers globally, and it needs further research to draw any conclusions and establish facts [13].

This study was conducted among 180 patients diagnosed with GSD. Women were predominant at 124 (69%) compared to men at 56 (31%). This finding is in accordance with the study conducted by Ghadhban et al. [14]. An evaluation of the thyroid profile was performed for each of the 180 patients, and the results revealed that 20 (11.11%) patients had clinical hypothyroidism, 35 (19.44%) patients had subclinical hypothyroidism, three (1.66%) patients had hyperthyroidism, and the other 122 (67.77%) patients were found to be euthyroid. In a study by Tombisana et al., it was determined that the age group of 46-55 years comprised the highest percentage of patients diagnosed with hypothyroidism, with 17 (39.5%) patients falling into this age range. Hypothyroidism was found in 29 (23.1%) of the 126 female patients who participated in the same study, whereas hyperthyroidism was found in six (4.7%) patients. When it came to male patients, there were 14 cases of hypothyroidism (18.9%) and three (3.9%) cases of hyperthyroidism [6]. These findings were consistent with the findings of our investigation.

Earlier observational studies reported a positive correlation between thyroid disorders and the development of GSD. A study by Inkinen et al. concluded that there was an association between hypothyroidism and the development of CBD stones and GSD [15]. Another study conducted by Völzke et al. [9] among 3,749 persons, aged 20 to 79, concluded that there was an independent correlation between GSD and increased levels of serum TSH. Nevertheless, no such correlation was found among female patients. Conversely, research has indicated that women are more prone to both GSD and thyroid issues [16]. In addition, patients with cholelithiasis showed a notably greater occurrence of both subclinical and clinical hypothyroidism [17].

In this study, dyslipidemia in hypothyroidism was significantly higher compared to euthyroid patients (p = 0.012). Marwaha et al. [18] concluded that people with hypothyroidism exhibited a significant increase in blood cholesterol and low-density lipoprotein (LDL) levels. Furthermore, Hussain et al. [19] demonstrated a substantial increase in total cholesterol, TG, and LDL levels in subclinical hypothyroid patients when compared to the control group. Furthermore, Pedrelli et al. [20] demonstrated elevated concentrations of total cholesterol and LDL in individuals with hypothyroidism. This can be attributed to the impact of thyroid hormone on the 3-hydroxy-3-methylglutaryl coenzyme A reductase enzyme as well as the effects of thyroxine on lipid metabolism in the liver. This finding is, however, in contradiction to Issa et al. who conducted a study among 232 patients, 175 patients with dyslipidemia, and found that only 25 had hypothyroidism, concluding that there was no correlation between hypothyroidism and cholelithiasis [21].

Limitations

The study was conducted at a single center. To validate the findings and provide recommendations for screening for thyroid dysfunction, a longer study duration with a larger sample must be conducted, which can be accomplished through a multicenter trial.

## Conclusions

In this study, the prevalence of hypothyroidism in GSD was found to be 30%, which is substantial. Subclinical hypothyroidism was identified in 19%, clinical hypothyroidism in 11%, and hyperthyroidism in 2%. This study also found a greater prevalence among females over the age of 40 years. Hypothyroidism is a specific risk factor for cholelithiasis, and all patients with GSD with dyslipidemia should be screened for thyroid dysfunction.
